# Building Capacity for Implementation Science in Precision Health and Society: Development of a Course for Professional and Graduate Students in Pharmacy

**DOI:** 10.3390/jpm12091499

**Published:** 2022-09-13

**Authors:** Megan C. Roberts, Jacqueline E. McLaughlin

**Affiliations:** 1 Division of Pharmaceutical Outcomes and Policy, UNC Eshelman School of Pharmacy, University of North Carolina at Chapel Hill, Chapel Hill, NC 27599, USA; 2 Center for Innovative Pharmacy Education and Research, UNC Eshelman School of Pharmacy, University of North Carolina at Chapel Hill, Chapel Hill, NC 27599, USA

**Keywords:** pharmacy, education, implementation science, precision health, course development, capacity building

## Abstract

Training in the field of implementation science is critical for future pharmacists and pharmaceutical scientists to successfully implement precision health interventions in pharmacy practice. We developed an elective course for second- and third-year students at the UNC Eshelman School of Pharmacy to develop foundational knowledge in implementation science with a focus on precision health implementation. The eight-week course used a flipped classroom format featuring lecture videos, suggested readings, quizzes, guest lectures from experts, case studies, and a group project. We evaluated course quality through class participation, a pre- and post-test on course content, and a mixed-methods survey completed by the students. Overall engagement in in the course was high and students demonstrated significant improvement in understanding of implementation science and precision health. Strengths of the course as identified by students were the use of expert guest speakers, pre-class lectures, and case study exercises, while the ordering of content and improved connection between content and guest lectures were identified as areas for improvement. In conclusion, the elective course was well-received and meets a critical need in the field of pharmacy to build implementation science capacity. Future work is needed to expand and refine education for the implementation of precision health for pharmacy professionals.

## 1. Introduction

Calls for capacity building for the implementation of precision medicine have largely remain unanswered [1,2]. One important component of this capacity building relates to educating health professionals and researchers in the field of implementation science to more effectively impact the adoption, implementation, and sustainability of precision health approaches in clinical practice.

In 2017, the field of pharmacy practice began recognizing the importance of training pharmacists and pharmaceutical science researchers in the field of implementation to advance pharmacy practice [3,4], noting a gap between the generation of new pharmaceutical knowledge and its translation into clinical practice. Implementation science offers a set of tools to promote the widespread uptake of existing and emerging evidence-based practices to improve medication therapy outcomes and delivery [4]. Implementation science training will be particularly important in the field of precision health which requires the implementation of highly complex interventions into practice, such as pharmacogenomics testing for precision dosing and selection. Implementation science is a transdisciplinary field and so the need for interprofessional learning may be particularly important [5].

To address this gap, we developed a course for second- and third-year Doctor of Pharmacy (PharmD) students and Doctor of Philosophy (Ph.D.) students across translational pharmaceutical science research pathways at the UNC Eshelman School of Pharmacy. First available in the spring of 2020, this eight-week course is taught every two years to provide foundational knowledge about implementation science with a focus on the implementation of precision health. The objective of this paper is to describe the design of the preliminary course and report on engagement, learning, and process outcomes from our first semester offering the course.

## 2. Materials and Methods

### 2.1. Course Description

This course was offered as an elective to second- and third-year students in the PharmD program as well as all students in the Ph.D. programs at the UNC Eshelman School of Pharmacy. One division, the Division of Pharmaceutical Outcomes and Policy, made the course a requirement for their PhD program. To accommodate the PharmD curriculum, the course was offered twice in the Spring of 2020 during two 8-week blocks for 2.5 h once a week. We used a flipped classroom format that included both pre-class and in-class activities. All new course content was delivered pre-class [6,7]. Pre-class activities included watching lecture videos with an introduction to new material and checks for understanding as well as suggested seminal readings (weeks 1–5, approximately 20-min per video). Pre-class lectures included the following 5 topics: introducing the field of implementation science in precision health; theories, frameworks, and conceptual models; implementation outcomes; implementation strategies; and implementation study designs. The remaining 3 weeks were dedicated to synthesizing and applying critical concepts (weeks 6–8). In-class activities included: (1) lecture quizzes using Poll Everywhere (San Francisco, CA, USA) to evaluate whether students watched and understood lecture videos (e.g., 5 multiple choice, fill in the blank, or open text questions), (2) review pre-class material based on lecture quiz results from prior week (approximately 10 min), (3) guest lecture from experts in the field with an emphasis on real-world application of pre-class materials (30–40 min lecture, 15 min discussion), (4) case study in small groups that allowed students to apply new material to a real research or practice problem in the literature (approximately 20–30 min), and (5) group project collaboration to apply implementation science to a project of the groups choosing (20–30 min per class period).

Learning objectives included:

1. Define implementation science.
2. Define precision health.
3. Summarize common challenges to the implementation of precision health in pharmacy practice.
4. Identify and describe implementation science frameworks, models, and theories that can be used to promote implementation of precision health in pharmacy practice.
5. Apply an implementation science framework, model, or theory to guide how one would tackle an implementation challenge.
6. Define and describe how implementation strategies can be employed to translate precision health into practice.
7. Select implementation strategies to overcome an implementation challenge in precision health research or pharmacy practice.
8. Describe the types of study design that are commonly used by implementation scientists.
9. List and define implementation science outcomes that can be measured to evaluate implementation.
10. Select which implementation outcomes could be measured to better understand the implementation of precision health in pharmacy practice.
11. Apply implementation science principles to a challenge in pharmacy practice or research.
12. Use implementation science to solve practice problems and/or to answer research questions.

Guest lecturers were asked to prepare a lecture describing how they have applied the pre-class content (e.g., implementation science frameworks) to a precision medicine research project or other pharmaceutical sciences project. In addition, they were asked to include at least 1 activity (e.g., think-pair-share) and time for discussion. Following each guest lecture, students completed a minute paper summarizing their main learning and their remaining questions [8]. These remaining questions were addressed during the review of pre-class material during the following class period.

### 2.2. Grading

Final course grades were based on the group project (0–45 points), course quizzes (0–25 points total), participation (0–20 points), and the group project proposal (0–10 points). Thus, this final score reflects both student engagement and learning outcomes. The group project served as the primary evaluation of student learning (see Supplemental Table S1 for rubric). Students were assigned to groups of 3 to 4 at the beginning of the course that purposefully included both PhD and PharmD students. Throughout the course, the students applied course content to an implementation question of the group’s choosing. Part way through the block during week 5, groups submitted project proposals for a grade; feedback was provided within a week. Group presentations of the project were delivered on the final day of class and all students were required to contribute to Q&A periods following presentations by raising at least one question or comment.

Class participation and course quizzes comprised the remainder of the course grades. For class participation, students could receive a maximum of 20 points for class participation, that is 2.5 points per class period. By attending class on time, students earned 2.0 points; however, 0.5 points could be added by asking at least 1 question to guest lecturers and 0.5 points could be lost for not following each of our class policies (e.g., use of phone or computer for non-class activities, unexcused tardiness, not participating in small group activities). Students received an update of this grade every 2 weeks (out of 5 points) to monitor their grade. The final course grade was determined on the following scale: fail, low pass, pass, high pass.

### 2.3. Course Evaluation Metrics

In order to evaluate this course, we measured student engagement and learning, as well as course processes.

Engagement. To assess engagement, we measured class participation and pre-class video access. Pre-class lecture videos were hosted via Panapto (Seattle, WA, USA), and data on the number of views were collected. We report cross-sectional data on the proportion of students who completed the videos.

Learning. In order to assess student comprehension, we administered a 10-question pre- and post-test that included open-ended and multiple-choice items that aligned to each learning objective. Of note, application objectives were tested via the final project, rather than through the pre-post test. We used a paired sample t-test to determine whether comprehension differed before and after the course. In addition, final course grades were evaluated with a goal for all students to pass the course (70/100 course points or higher).

Process. Through course evaluations, we collected quantitative and written qualitative data on student perceptions of the course organization, in-class activities, assessments, whether the class was challenging, and overall (see Table 1). Written qualitative data were centered around (1) course strengths, (2) “what helped you learn/was motivational/was valued/useful?” (3) “what would make the course a better learning experience? and (4) “what hindered your learning/was demotivating/was not valued/useful?” Open-ended responses were summarized by one coder, who iteratively reviewed all open-ended responses, developed a list of emergent themes, and applied the final code set to all open-ended responses.

As this course was taught in back-to-back 8-week blocks, there was an opportunity to make some rapid adaptations for Block 2 based on student feedback from Block 1. These adaptations were documented systematically and assessed through student course evaluations. In addition, adaptations were made in the course due to COVID-19, including moving the course to a fully online platform. These changes are also described below in the results. We present descriptive statistics (means, medians) and compare process outcomes between Blocks 1 and 2 using a *t*-test.

This study was reviewed by the Office of Human Research Ethics, which has determined that this submission does not constitute human subject research as defined under federal regulations [45 CFR 46.102 (e or l) and 21 CFR 56.102(c)(e)(l)] and does not require approval.

## 3. Results

In total, 17 students were enrolled in the elective course over Blocks 1 (*n* = 10) and 2 (*n* = 7). Overall, six were PhD students from three of the school’s five divisions (Divisions of Pharmaceutical Outcomes and Policy, Pharmacoengineering and Molecular Pharmaceutics, and Pharmacotherapy and Experimental Therapeutics), and 11 were PharmD students in their second year of study. 

### 3.1. Engagement

**Figure 1:**
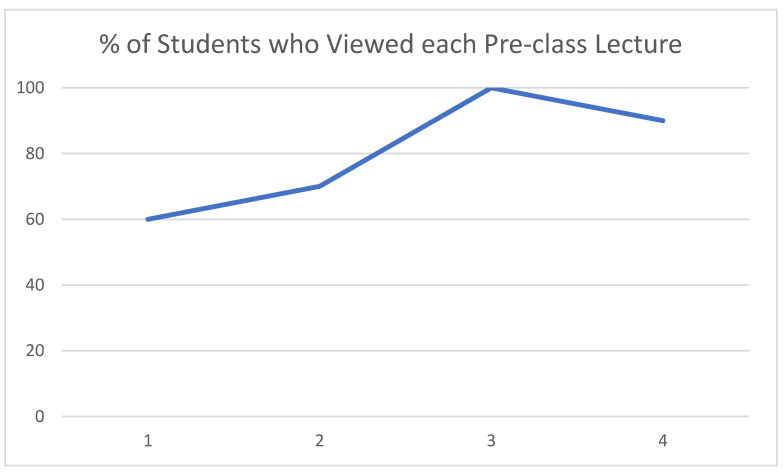
Pre-class lecture engagement.

Overall engagement was high. On average, final class participation was 96.5% and 94.5% for Blocks 1 and 2, respectively. Views of pre-class videos increased over time, from 60% participation for lecture one to 90% participation by lecture four during Block 1 (Figure 1). Due to a technical error, findings from Block 2 are not available.

### 3.2. Learning

The mean final grade average was 92.2% (median = 92, standard deviation = 2.51). We found significant improvements in knowledge of implementation science and precision health and society from before and after the course (Block 1: 65% pre, 92.9% post, *p* < 0.01; Block 2: 73.6% pre, 90.4% post, *p* < 0.01).

### 3.3. Process

Overall course feedback can be found in Table 1. On average, students highly rated the organization, activities, assessments, and intellectual challenge of the course. Feedback on the course was elicited halfway through each block and changes were made in real time. Major changes resulting from this feedback included: (1) showing correct answers in practice questions that were embedded in pre-class videos, (2) spending more time connecting pre-class content to in-class content at the beginning of class, and (3) transitioning to an all-online format with breakout rooms partway through Block 2 due to COVID-19. During this time, all lectures were recorded and available upon request. Improvements were made between blocks, and students more strongly endorsed that “the course challenged me to think deeply about the subject matter” (*p* = 0.046) in Block 2 compared to Block 1.

**Table 1:** Course Evaluation (*n* = 17).

Evaluation Item [never, 1, to always, 5]	Overall		Section 1		Section 2		*p*
	n = 17		n = 10		n = 7		
	Mean	Median	Mean	Median	Mean	Median	
*This course was well organized.*	4.71	5.00	4.60	5.00	4.86	5.00	0.57
*The in-class activities/exercises contributed to my learning.*	4.35	4.00	4.30	4.50	4.43	4.00	0.70
*The assessments were clearly connected to the course outcomes.*	4.50	5.00	4.30	5.00	4.83	5.00	0.26
*This course challenged me to think deeply about the subject matter.*	4.12	4.00	3.80	4.00	4.57	5.00	0.046
*Overall rating [poor, 1, to excellent, 5]*	3.59	4.00	3.50	4.00	3.71	4.00	0.57

We also obtained qualitative feedback from students at the end of each block in order to understand student perceptions of course (Table 2). A number of strengths were noted about the structure of the class, including the use of (1) expert guest speakers, (2) cases for in-class exercises, and (3) pre-class lectures. In addition, some recommendations were given including reordering the lectures and improving connections between guest lectures and the pre-class lectures.

## 4. Discussion

As a field, pharmacy has recognized the importance of capacity building for implementation science among pharmacists, researchers, and faculty [3,4]. More recently, attention to the importance of incorporating implementation science into the pharmacy curriculum has been highlighted by the American Association of Colleges of Pharmacy [9,10]; however, to date, the development of such courses has been limited. To our knowledge, this is the first report outlining the application of implementation science in pharmacy education. Other health fields, such as public health, nursing, and social work, have advanced the incorporation of implementation science for the training of both researchers and practitioners [11,12,13,14,15]. This course represents one step towards addressing calls for incorporating implementation science to advance pharmacy practice. In particular, given the increasing complexity of implementation in the era of precision health, offering a course in implementation science with a focus on precision health advances not only needs in the pharmacy space, but also precision medicine more broadly [1,5].

Overall, this course was well received among PharmD and PhD students. In particular, the structure of the flipped classroom was well received, and students enjoyed the opportunity to work within transdisciplinary groups to complete case studies and their final projects. By partnering PharmD and PhD students, peer learning allowed for students to gain clinical and research perspectives. Final projects demonstrated students’ ability to apply implementation science methods to address real-world implementation challenges in precision health. These skills will be essential for clinicians and precision health researchers alike, as precision approaches are increasingly being translated into clinical settings. In addition to final projects, guest lectures by practitioners and researchers in the field provided real-world examples to the students of how implementation science can change practice and improve the integration of new precision health technologies to benefit patient health.

Some challenges emerged from the course. Firstly, more foundational content needs to be introduced ahead of teaching the students about conceptual frameworks to guide implementation processes, evaluation, and identifying determinants. To this end, additional material will be provided in the introductory in-class lecture on day one to prepare students for future lectures on frameworks, outcomes, strategies, and study designs. Secondly, students felt that implementation science jargon was not well defined and understood. Future semesters will include a “vocab of the day” list at the beginning of pre-class lecture videos and at the start of the subsequent class period, and these terms will be reviewed and applied to examples. Finally, more connection between pre-class content and guest lectures was requested. To improve this, moving forward, guest lecturers will be asked to submit slides one week in advance so that the course director can provide an introduction to their lecture that connects pre-class lecture material to their talk. 

This evaluation has several limitations. Firstly, this comes from a single institution and has a limited sample size, which limits the generalizability of findings. We lacked a control group as this is the first time the course has been offered. All quantitative course evaluation data were provided in aggregate, so we were unable to calculate interquartile ranges. Finally, this is exploratory in nature. More research is needed to explicate the impact of course activities and changes on engagement, learning, and process outcomes.

As precision health technologies increasingly become mainstream, health care professionals, including pharmacists, must be engaged in transdisciplinary teams to identify and address implementation barriers. In the absence of this, we run the risk of worsening existing disparities in patient care [5].

## Figures and Tables

**Table 2:** Qualitative answers about the course structure and processes (*n* = 17).

Strengths	Exemplar Quotes
Structure: guest speakers (*n* = 11)	*The course brought in a lot of experts in the field to talk about their research and experience in the field. They brought in new points of view that kept the class changing.*
Foundational nature (*n* = 5)	*The course introduced implementation science, a fresh area to me, and the research methods introduced in class could help me conduct research on pharmacy practice/health service.*
Structure: introduction to new material to application (*n* = 2)	*I really liked how the course was structured, especially with the focus on foundational topics at the beginning, and then transitioning to application of those topics later in the course.*
Structure: class cases (*n* = 10)	*I feel like I learned a lot in this class. Some of the concepts are still a little abstract to me but I think I have built a foundation on what is implementation science. I like the in class cases as they allowed me to really put in the work and understand the class materials in a more applicable way.*
Structure: pre-course materials (*n* = 8)	*I appreciate that the course introduced us to various health science disciplines so that we could understand how implementation science informs research across different disciplines. I also found the pre–class modules to be helpful in teaching me important foundational concepts prior to class.*
Structure: pre-course materials (quizzes) (*n* = 1)	*…the quizzes at the end of each video are good ways to check my knowledge.*
Structure: QA (*n* = 2)	*I liked the group discussions on the various topics.*
Structure: course-long project (*n* = 1)	*I like how we worked on the project throughout the course of the semester. It made it very valuable to continue learning and applying knowledge.*
**Weaknesses**	
Order of concepts: frameworks later (*n* = 5)	*I think some of the course information could have been re–ordered to make the material easier to understand. I think leading with the more abstract principals like frameworks before discussing how they’re applied can be confusing.*
Linking IS concepts (*n* = 1)	*...the course did not do a good job of linking the different topics together until later in the semester so a majority of the time I was a little lost.*
Linking guest speaker content to pre-class material (*n* = 3)	*Hard to apply the guest speaker information into the context of the class.*
Preparation of guest speakers (*n* = 1)	*I felt like some of the presenters who came in were not really prepared to present*
IS terminology (*n* = 3)	*Sometimes I knew what words I was supposed to use in the class, but I had no idea what that meant in practice.*
PowerPoints for pre-class videos available (*n* = 2)	*I would have liked the lecture slides from the pre–class videos to be available on sakai.*
Pre-class self quiz banks (*n* = 2)	*For the first 3–4 quizzes, the pre–class material did not give feedback on the assessment. [Block 1]*
Shorter class period (*n* = 2)	*I felt like the length of the class period partially hindered learning. It was difficult to stay engaged for the full 2.5 h.*
Make course a full semester (*n* = 1)	*I think having this course throughout a full semester would help because this is a very broad subject and can be very specialized, so it’s hard to learn in a few weeks.*
More discussion of cases (*n* = 1)	*I would like more case discussion and more deep explanations about the research methods, especially the qualitative research.*
More practice before cases (*n* = 1)	*Some of the in–class exercises felt abstract… maybe because we had just learned the material.*
Move pharmacy-specific guest lectures to beginning (*n* = 1)	*I think it might be beneficial for students if the guest lectures in the beginning of the course are more focused to the intersection between implementation science and pharmacists.*
No need for longer guest speaker seminars at end of Block 1 (*n* = 1)	*The long [guest] lectures in the last 3 weeks didn’t help much.*
Move cases and speakers to second half of block (*n* = 1)	*think that the course needs to focus on learning the basics for the first half of the semester then bring in the case reports and guest speakers.*

## Data Availability

The data presented in this study are available on request from the corresponding author. The data are not publicly available to protect student confidentiality given the small sample size.

## Supplementary Materials

The following supporting information can be downloaded at: https://www.mdpi.com/article/10.3390/jpm12091499/s1, Table S1: Final Project Rubric.
