# Supplementary material for: Building Capacity for Implementation Science in Precision Health and Society: Development of a Course for Professional and Graduate Students in Pharmacy
Source: J Pers Med. 2022 Sep 13;12(9):1499. doi: 10.3390/jpm12091499 (PMC9502747; doi:10.3390/jpm12091499)
Supplement: Supplementary file 1 [file jpm-12-01499-s001.zip › jpm-1813073-Supplementary.pdf]

Table S1: Final Project Rubric.

The goal of this project is to apply principles of implementation science to a phramacy practice or research challenge of your group's choosing. Principles that will be assessed are listed below. We will also do an assessment of group participation (by your group-peers).				
**Project Component**	**Total Points (45 pt**	**Maximum Points**	**Partial points**	**No points**
**Define and provide a rationale for the implementation gap/challenge that your project addresses**	**5**	The implementation challenge is well described. All needed background information is provided. The implementation gap has been well defined and the need for the implementation study has been clearly rationalized with data from existing literature.	The implementation challenge is described, but perhaps not clear or with complete background information. Because of this, the rationale for studying this implementation gap is discussed but not completely clear.	The implementation gap studied is not justified. Data and background material is not presented.
**Define the evidence-based practice**	**2**	The evidence-based practice that is being implemented is clearly described.	The evidence-based practice was defined but not clearly.	The evidence-based practice was undefined.
**Define the objective**	**3**	The objective of the group's study is well defined (SMART goal)	The study objective is described and meets the goals of the assignment, but remains unclear.	The study objective was not presented or the objective does not align with the goals of the assignment (to design an implementation science project)
**Select an implementation framework, theory or model and describe why it was chosen and how it was used.**	**5**	Implementation framework, model, or conceptual model was identified to guide the study. The rationale for selecting this framework is well described and fits the objective of the project.	Implementation framework, model, or conceptual model was identified to guide the study. The rationale for selecting this framework was not fully described and/or it is not fully clear how the framework model or conceptual model aligns with the objective of the project.	No implementation science framework, model, or conceptual model was identified to guide the study.
**Describe use of implementation strategies and provide a rationale**	**5**	Implementation strategies are described and rationale for selecting these strategies is described. If no strategies are leveraged, the rationale is described and is clear.	Implementation strategies are described but the rationale for selecting these strategies is not fully clear.	Implementation strategies are either not discussed and/or no rationale is provide for the selection of used strategies.
**Select, define and provide a rational for implementation outcomes**	**5**	Implementation outcomes are measured and defined. A rationale for choosing to measure the selected outcomes is provided. How these outcomes will be measured are well outlined and where possible, measures are pulled from the literature.	Implementation outcomes are discussed, however the definition, measurement and rationale for each outcome was not fully clear.	Implementation outcomes were either not included in the study or were not described.
**Describe the study design**	**5**	The study design is described in detail, and a clear rationale for choosing this design is provided.	The study design is not fully described in detail, and/or the rationale for choosing this design is not fully clear.	The study design was not discussed or did not align with the goals of the assignment.
**Describe the study context**	**5**	The study context is clear: The study setting (e.g., UNC Hospital) is defined as well as the patient population. The who, what, where, and when has been outlined clearly.	The study context was described, but aspects were not fully clear, including the study setting and participants (who, what, where, when).	The study context was not described.
**Provide a high quality presentation**	**5**	Quality of the presentation was superior. The slides are clear, and well organized. The talk was clearly rehearsed and professional.	Quality of the presentation was average. There were basic errors and it is unclear whether the team practiced their talk.	The quality of the presetnation was poor. There were basic errors throughout the presentation and/or slides are unorganized. Presentation was unprofessional.
**Complete group feedback form**	**5**	Full points for group participation score. It is clear that the individual participating fully in the final presentation.	The individual did not fully participate in the final presentation.	If there is evidence that an individual did not contribute at all to the final presentation, Dr. Roberts will discuss with the student and group. Note: failing to participate will compromise an individual's final grade, even if the group attained points in other component areas.
